# System-Wide Immunohistochemical Analysis of Protein Co-Localization

**DOI:** 10.1371/journal.pone.0032043

**Published:** 2012-02-21

**Authors:** MinJung Kim, Virawudh Soontornniyomkij, Baohu Ji, Xianjin Zhou

**Affiliations:** Department of Psychiatry, University of California San Diego, La Jolla, California, United States of America; University of Turin, Italy

## Abstract

**Background:**

The analysis of co-localized protein expression in a tissue section is often conducted with immunofluorescence histochemical staining which is typically visualized in localized regions. On the other hand, chromogenic immunohistochemical staining, in general, is not suitable for the detection of protein co-localization. Here, we developed a new protocol, based on chromogenic immunohistochemical stain, for system-wide detection of protein co-localization and differential expression.

**Methodology/Principal Findings:**

In combination with a removable chromogenic stain, an efficient antibody stripping method was developed to enable sequential immunostaining with different primary antibodies regardless of antibody's host species. Sections were scanned after each staining, and the images were superimposed together for the detection of protein co-localization and differential expression. As a proof of principle, differential expression and co-localization of glutamic acid decarboxylase67 (GAD67) and parvalbumin proteins was examined in mouse cortex.

**Conclusions/Significance:**

All parvalbumin-containing neurons express GAD67 protein, and GAD67-positive neurons that do not express parvalbumin were readily visualized from thousands of other neurons across mouse cortex. The method provided a global view of protein co-localization as well as differential expression across an entire tissue section. Repeated use of the same section could combine assessments of co-localization and differential expression of multiple proteins.

## Introduction

Cellular functions are determined by genome-wide gene expression, co-localization, and interactions of multiple proteins. Immunofluorescence histochemical staining is the most common method for the detection of protein co-localization. However, there are several limitations for the method. First, primary antibodies need to come from different species, and fluorescence signals are susceptible to photobleaching. Second, it can be difficult to conduct immunofluorescence histochemical staining on paraffin sections due to high levels of autofluorescence. Third, co-localization of fluorescent signals is often examined in localized areas due to lack of high-resolution scan across a whole tissue section. On the other hand, chromogenic immunohistochemical analysis is, in general, not suitable for the analysis of protein co-localization. The development of multi-target chromogenic immunohistochemistry partially alleviated the problem [1]. However, it still requires primary antibodies coming from different species. Furthermore, only a limited number of commercial antibodies are suitable for immunohistochemical analysis on paraffin sections. Here, we describe a sequential method for chromogenic immunohistochemistry to study protein co-localization and differential expression on paraffin sections. After scanning the sections, the images from different antibody staining can be superimposed to visualize protein co-localization as well as differential expression across an entire tissue section. The method should be useful for those experiments where paraffin sections are needed for superior cell morphology and for human pathological studies where most tissues are preserved on paraffin sections. As a proof of principle, we conducted chromogenic immunohistochemical analysis of co-localization and differential expression of glutamic acid decarboxylase67 (GAD67) and parvalbumin proteins in mouse cortex.

## Results

**Figure 1:**
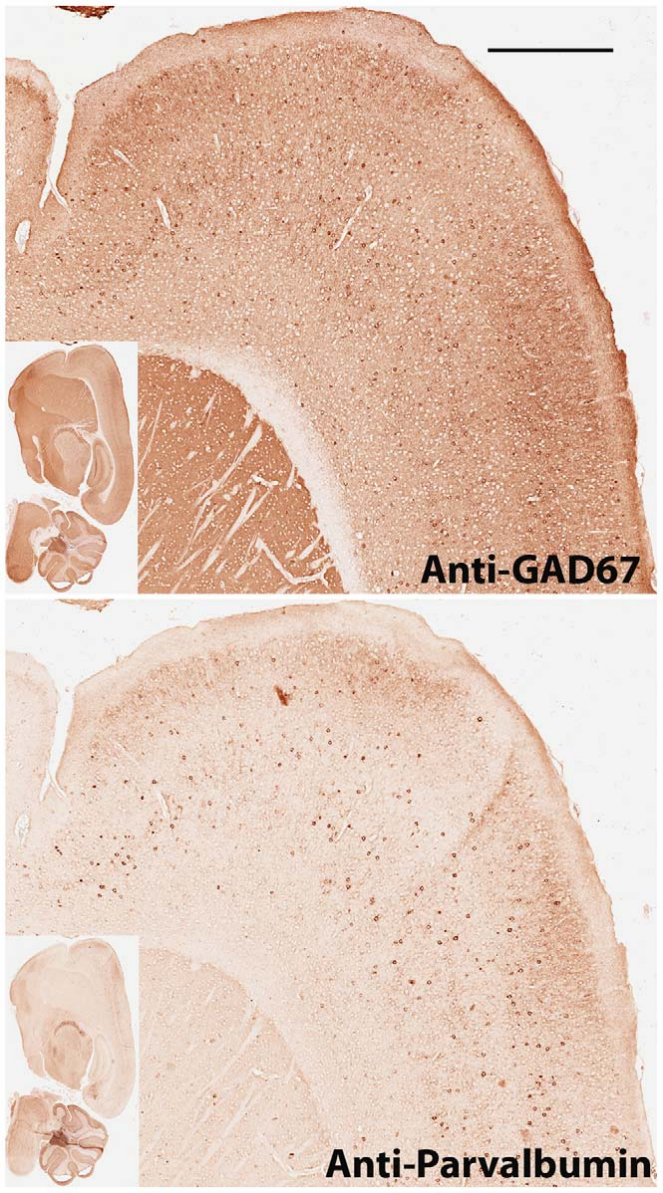
Chromogenic immunohistochemical analysis of GAD67 and parvalbumin in mouse cortex. Mouse brain paraffin section was first used for anti-GAD67 immunostaining. After the stained slide was scanned with Aperio ScanScope, the anti-GAD67 primary antibody and red stain color were completely stripped. The same section was re-used for anti-parvalbumin immunostaining.

**Figure 2:**
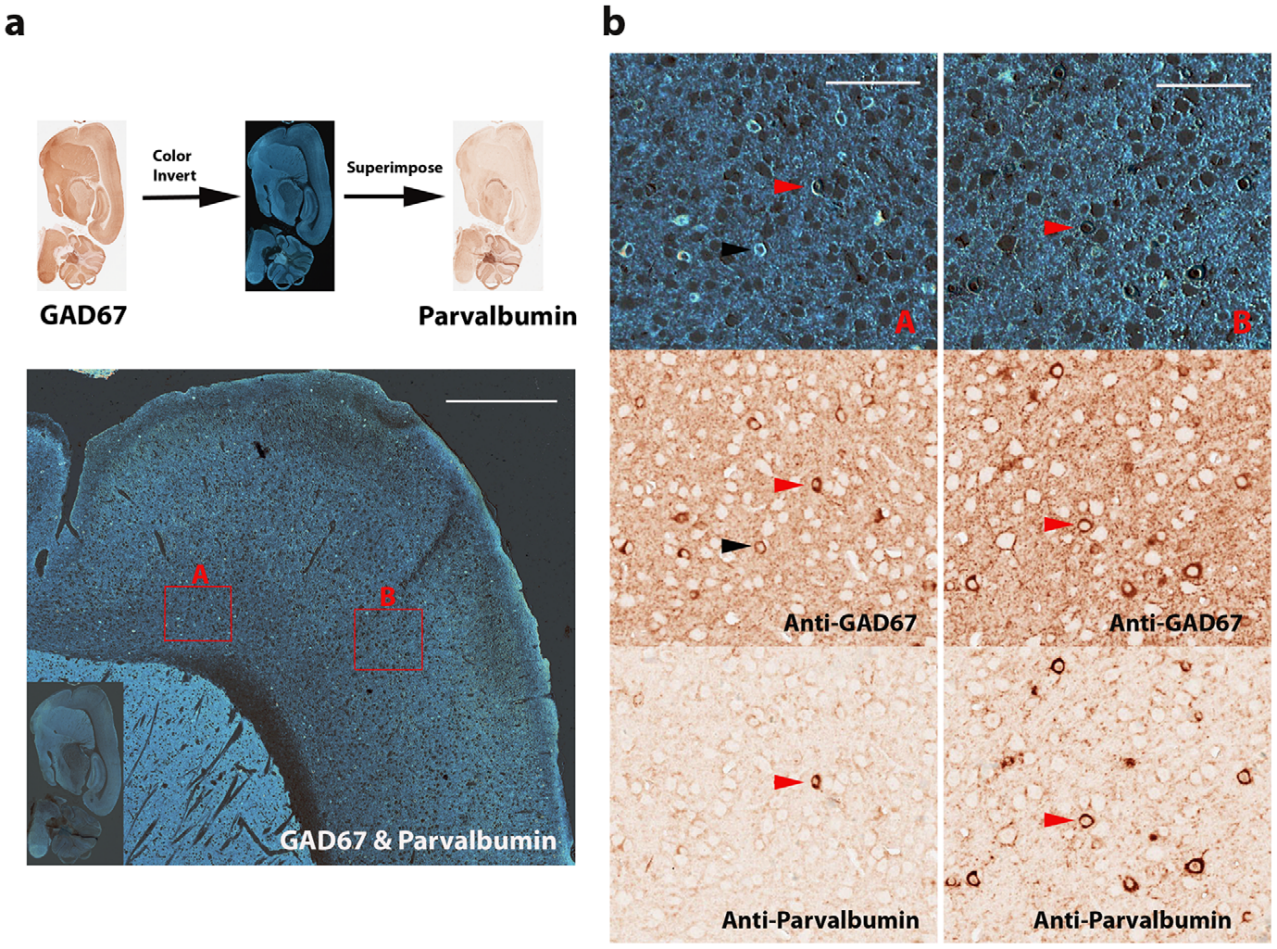
Differential expression of GAD67 and its co-localization with parvalbumin. (**a**) The color of the anti-GAD67 image was inverted to generate bright GAD67 signal against a dark background. The inverted image was further superimposed onto the anti-parvalbumin image (bottom). GAD67 expression only cells, mostly in superficial and deep layers of cortex, were observed as bright spots. Scale bar: 500 µm. Deep layers (A) and middle layers (B) were visualized at a higher magnification (**b**). GAD67 expression only neurons and its co-localization with parvalbumin in the deep layers (A) of frontal cortex and the middle layers (B) of motor cortex. Red arrow heads pointed to neurons expressing both GAD67 and parvalbumin; black arrow heads pointed to neurons expressing GAD67 only. Scale bar: 100 µm.

**Figure 3:**
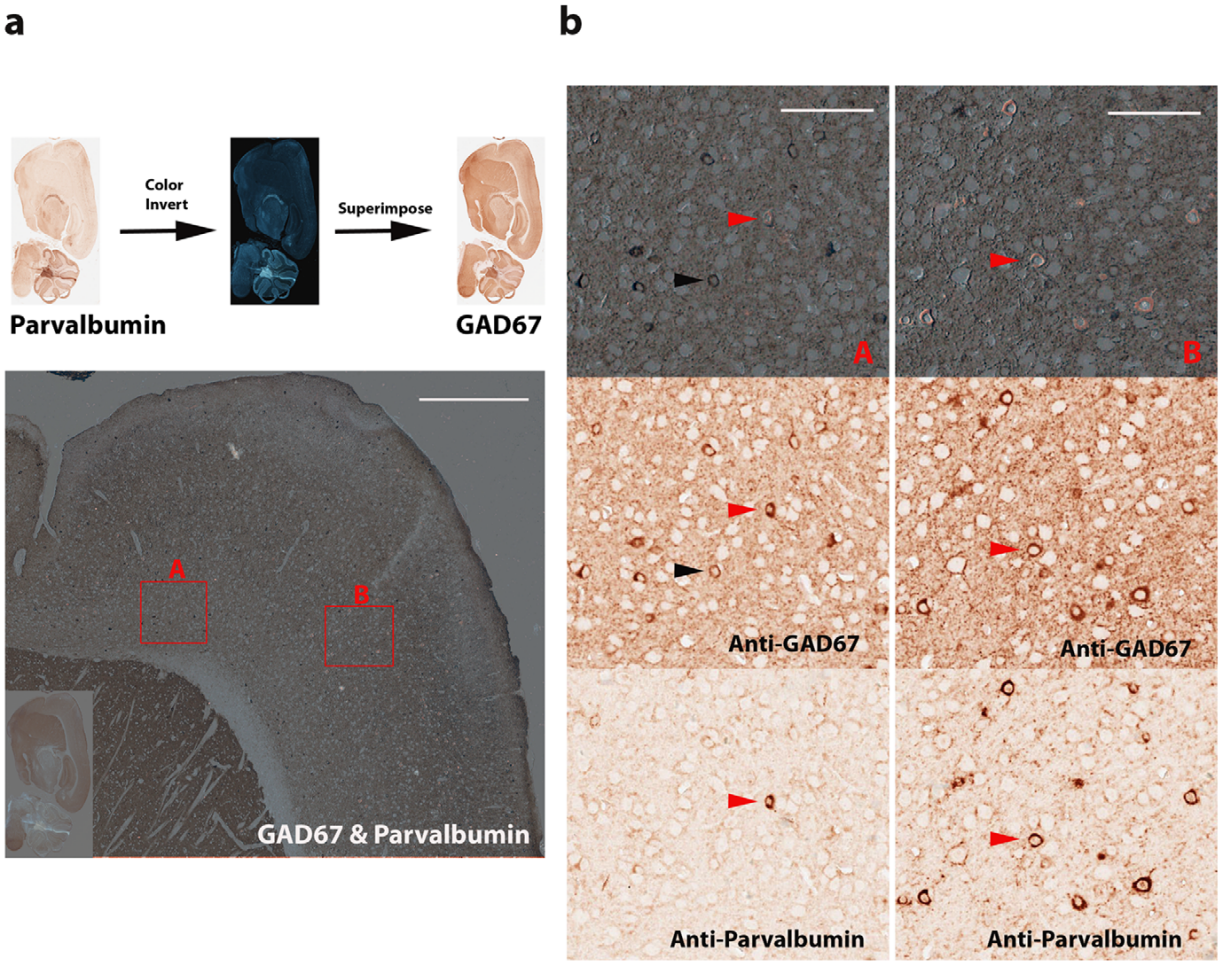
Parvalbumin and GAD67 co-localization. (**a**) The color of the anti-parvalbumin image was inverted and further superimposed onto the anti-GAD67 image (bottom). Parvalbumin expression only cells would appear as bright spots. However, there were no parvalbumin-positive cells that did not express GAD67. Scale bar: 500 µm. Deep layers (A) and the middle layers (B) were visualized at a higher magnification (**b**). GAD67 expression only neurons became darker spots in deep layers (A) of frontal cortex (black arrow heads). Co-localization between GAD67 and parvalbumin in the middle layers (B) of motor cortex (red arrow heads). Scale bar: 100 µm.

**Figure 4:**
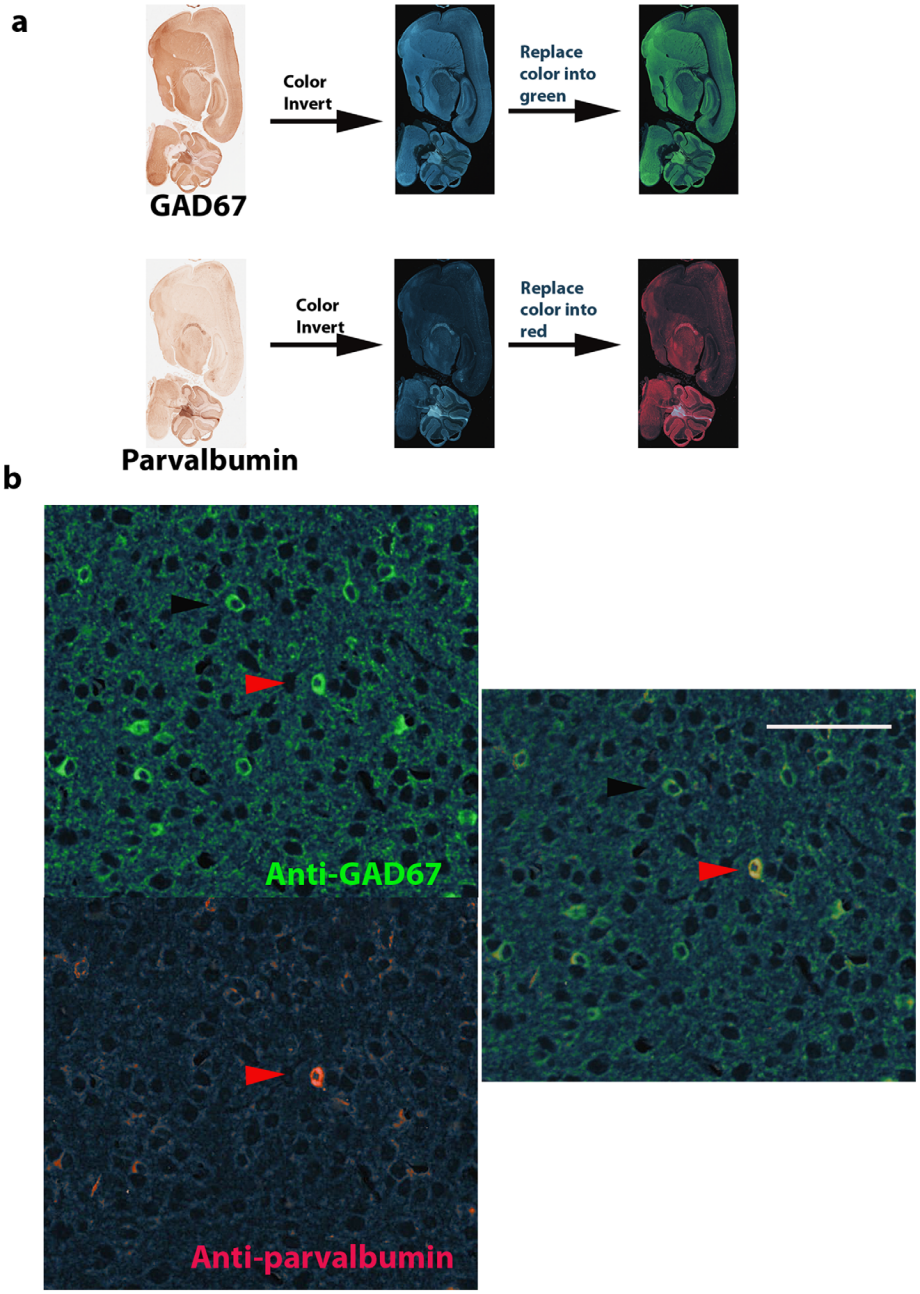
Co-localization between GAD67 and parvalbumin in two different colors. The color of both anti-GAD67 and anti-parvalbumin images was inverted. The bright signal in the inverted GAD67 and parvalbumin images was further replaced with green and red colors respectively (**a**). After being superimposed together, neuron expressing both GAD67 (green) and parvalbumin (red) became yellow (red arrow head)(**b**). Scale bar: 100 µm.

In mouse cortex, GABAergic interneurons expressing GAD67 consist of several different subgroups of neurons which express parvalbumin, calretinin, and somatostatin respectively [2]. Parvalbumin-positive neurons constitute the largest subgroup of GAD67 expressing interneurons. Mouse monoclonal anti-GAD67 was first used for chromogenic immunohistochemical stain with NOVA Red peroxidase substrate. Hundreds of GAD67 positive neurons were visualized with countless stained fine particles, presumably the clusters of presynaptic localized GAD67 [3], in mouse cortex (Figure 1). The brain section was scanned at 200× magnification (resolution: 0.5 micrometer/pixel) with Aperio ScanScope. After scanning image, the NOVA Red was completely removed in subsequent section rehydration, antigen retrieval, and stripping of the primary antibody. To ensure that the primary anti-GAD67 antibody was removed completely, we incubated the slide with the peroxidase-conjugated secondary antibody and conducted NOVA Red stain later. No red stain was observed, suggesting that the primary antibody was removed completely with our protocol (data not shown). The slide was subsequently re-used for staining with a next primary antibody, mouse monoclonal anti-parvalbumin. The expression of parvalbumin can be readily observed across mouse cortex (Figure 1). The slide was scanned again with Aperio ScanScope. To visualize co-localization and differential expression between GAD67 and parvalbumin proteins, we conducted color inversion of the GAD67 image. The GAD67 signal became bright against a dark background in the inverted image which was further superimposed onto the parvalbumin image (Figure 2a). The bright GAD67 expressing cells were dimmed to be invisible in the dark background when its expression was co-localized with dark red color of parvalbumin from the same cells. Sporadic GAD67 positive bright cells were readily visualized from the dark background across mouse cortex when little or no parvalbumin was expressed from the same cells. These bright GAD67 cells mostly came from either superficial layers (Figure S1) or deeper layers, fewer from middle layers of mouse cortex. To illustrate their co-localization and differential expression, both deep layers (A) and middle layers (B) of the mouse cortex were examined at a higher magnification (Figure 2a, 2b). The shortcoming for the above analysis is that cells expressing only parvalbumin may be hard to detect against the dark background at lower magnification. To visualize these cells expressing only parvalbumin in mouse cortex, we inverted the color of the anti-parvalbumin image to generate a bright signal for parvalbumin-positive cells against a dark background and superimposed it onto the GAD67 image (Figure 3a). Cells expressing only parvalbumin would become “bright spots” if no GAD67 was expressed. Except for a couple of artifactual “bright spots” (easily recognized under a higher magnification), all parvalbumin-positive cells also express GAD67. The co-localization and differential expression between GAD67 and parvalbumin in the regions A and B can also be confirmed at a higher magnification (Figure 3b). In both analyses, we took the same strategy to highlight differential expression against a dark background so that a system-wide differential expression could be visualized readily at a lower magnification across the entire mouse cortex. The candidate regions can be further examined under a higher magnification for confirmation. To complement the two above analyses where the co-localization dimmed cells were invisible, we took another approach to highlight GAD67 and parvalbumin signals into different colors. The color of both GAD67 and parvalbumin images was inverted, and their bright signals were further replaced with green and red colors respectively (Figure 4a). After being superimposed together, cells expressing both GAD67 and parvalbumin produced bright yellow color, and were readily recognized in mouse cortex (Figure 4b).

During the development of the above protocol, we noticed that different methods were published for antibody stripping [4], [5]. Therefore, we compared our stripping protocol with two other stripping methods using either KMnO4 [4] or Glycine/SDS [5]. Three slides were first used for immunohistochemical staining of GAD67 proteins (Figure S2). After the slide scanning, the GAD67 stain and antibodies were stripped from individual slides with each of the three methods. All three methods were effective in stripping since no significant staining was observed after further incubating the slides with the peroxidase-conjugated secondary antibodies and subsequent NOVA red reaction. However, only the slide stripped with our protocol successfully produced immunohistochemical staining of parvalbumin in the next round (Figure S2). We do not know why the two other methods failed, and suspect that parvalbumin antigen might have been modified by the acidic stripping solution containing either KMnO4 or Glycine/SDS.

**Figure 5:**
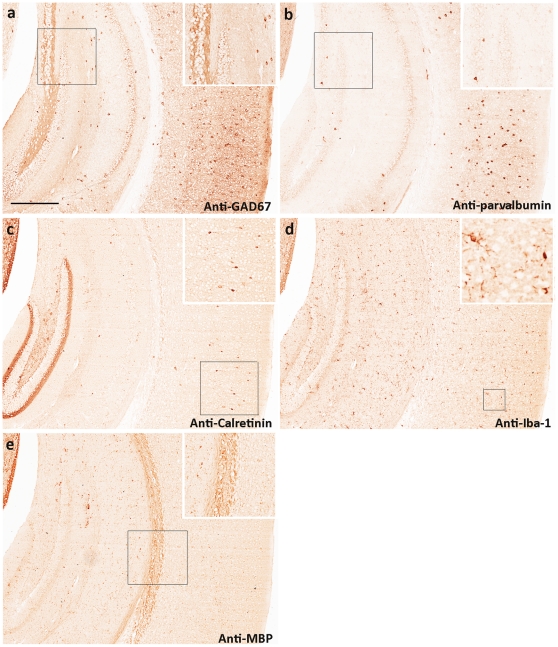
Multiple rounds of sequential immunohistochemical analysis. Mouse hippocampus and cortex were immunostained with 5 different antibodies: (**a**) anti-GAD67, high expression of GAD67 in hippocampal mossy fiber besides in cortex, (**b**) anti-parvalbumin, no detectable parvalbumin in hippocampal mossy fiber, but abundant in cortex, (**c**) anti-calretinin, sporadic calretinin positive cells in cortex with abundant calretinin expression in the inner molecular layer of dentate gyrus, (**d**) anti-Iba-1, numerous microglial cells and their processes across the section, (**e**) anti-MBP, abundant myelin in *corpus callosum* and some myelinated neurons in hippocampus and cortex. Scale bar: 500 µm.

**Figure 6:**
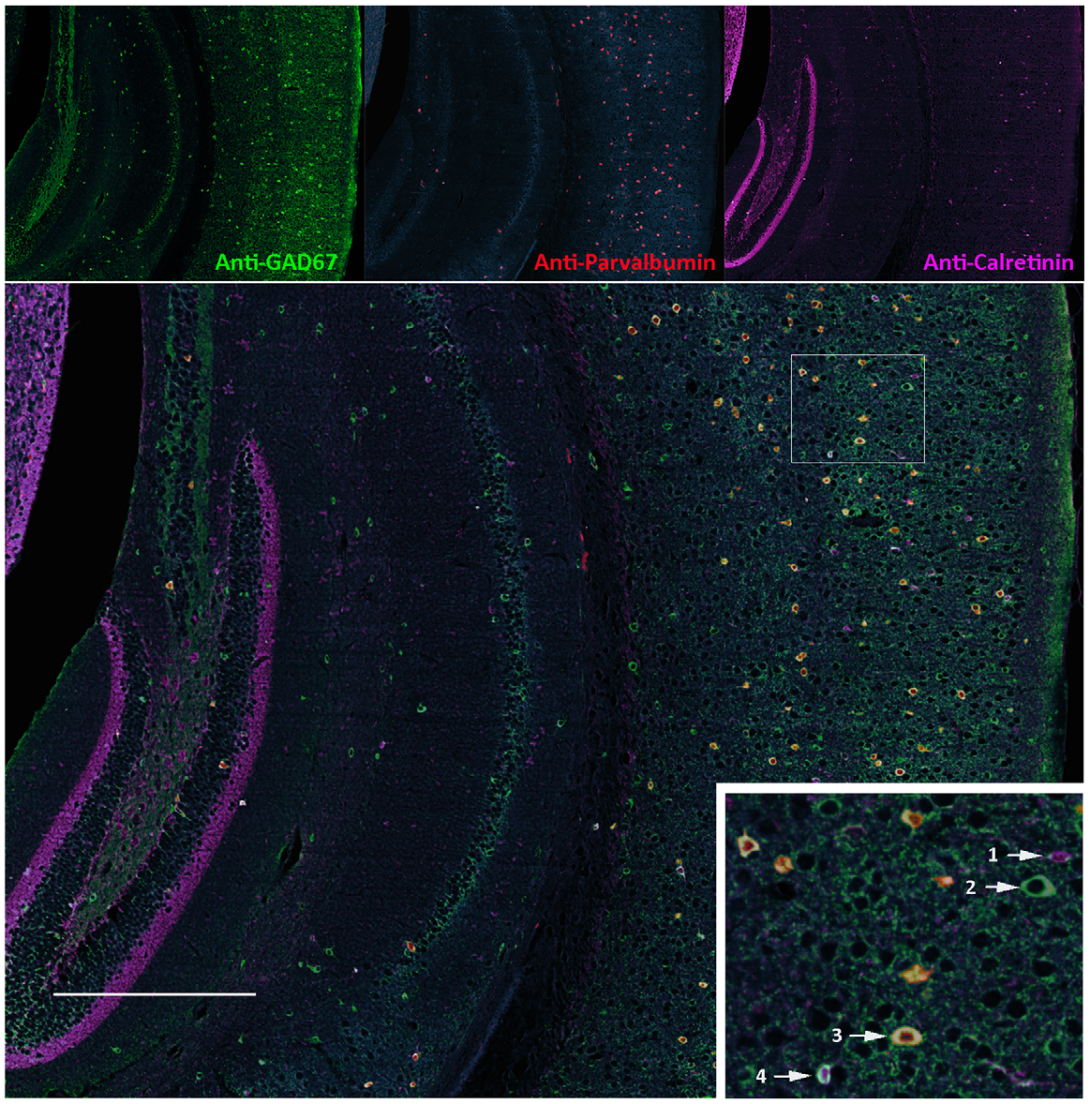
Co-localization between GAD67, parvalbumin, and calretinin. Top panel: the images of GAD67, parvalbumin, and calretinin were pseudocolored into green, red, and purple respectively. Bottom panel: the three images were superimposed together. Arrow 1: calretinin positive neuron. Arrow 2: GAD67 positive neuron. Arrow 3: neuron expressing both GAD67 and parvalbumin. Arrow 4: neuron expressing both GAD67 and calretinin. Scale bar: 500 µm.

To further evaluate robustness of our protocol, we conducted multiple rounds of stripping and immunostaining with different primary antibodies. Five different antibodies (anti-GAD67, anti-parvalbumin, anti-calretinin, anti-Iba-1, and anti-MBP) were used for sequential chromogenic immunohistochemistry. These antibodies have their distinct staining patterns across mouse cortex and hippocampus to help distinguish specificity and carry-over contamination. As expected, GAD67 has high expression in hippocampal mossy fiber (Figure 5a). After stripping, no carry-over (in mossy fiber) was detected in the second round of staining with anti-parvalbumin (Figure 5b). Numerous strong stains of parvalbumin positive neurons did not carry over into the third round of staining with anti-calretinin which detected much fewer cortical neurons (Figure 5c). Consistent with literature, high expression of calretinin was specifically observed in the inner molecular layer of dentate gyrus [6]. Anti-Iba-1 antibody successfully detected microglial cells and their processes across mouse cortex and hippocampus (Figure 5d). The anti-MBP was used as the fifth antibody which reacted with myelin in *Corpus Callosum* and gray matter (Figure 5e). After 5 rounds of stripping and immunostaining, the same section was used for two more rounds of immunohistochemical staining (data not shown). However, the section started to detach at microscopic level and generate higher background. To better illustrate distinct protein expression patterns and their co-localization, the images were converted into different pseudocolors and superimposed together. Our analysis demonstrated that calretinin and parvalbumin were never co-localized with each other in mouse cortex (Figure S3), supporting again the absence of any carry-over contamination. After superimposing the images of GAD67, parvalbumin, and calretinin, we generated a comprehensive cortical map of different GABAergic inhibitory neurons which were readily visualized by co-localization between GAD67 and parvalbumin or calretinin (Figure 6).

## Discussion

Protein co-localization is typically conducted on thicker cryosections with immunofluorescence histochemical staining, and can be visualized at high resolution with a confocal microscope. However, there are several limitations to these methods. Alternative approaches for protein co-localization analysis, particularly for paraffin sections, were developed with chromogenic immunohistochemistry [1]. However, each method requires a co-incubation of different primary antibodies coming from different host species. Additionally, the simultaneous co-incubation may not be feasible if antigens require different retrieval protocols. In this report, we developed a new method to completely strip previous primary antibody to ensure multi-target chromogenic immunohistochemical analysis on the same paraffin section. By taking advantage of physical robustness of paraffin sections, the same section can be re-used multiple times with different antibodies. No tissue deformation was observed during our first five rounds of stripping and immunostaining, although further processing started to cause some detaching within the section. With the optimization of the stripping conditions and the selection of glass slides to attach sections more firmly, it is likely that more rounds of sequential immunohistochemical staining can be conducted on the same section. In combination with a high resolution scan (up to 0.25 µm/pixel), a gigabyte size image of an entire brain section was obtained for each staining. Different approaches were explored to highlight either differential expression or co-localization against a dark background so that the objects of interest could be readily detected at lower magnification from mouse cortex. In our analysis, the differential expression and co-localization between GAD67 and parvalbumin were readily visualized. Consistent with previous reports, we did not find any parvalbumin-positive cells that did not express GAD67. However, there are a number of GAD67-positive cells expressing no or little parvalbumin, for example, some GAD67-positive neurons express calretinin instead of parvalbumin in our co-localization analysis. Such a system-wide analysis of protein co-localization and differential expression might be particularly useful for a quick global identification of any abnormal protein localizations in mutant mouse brain. Our method could also be useful for multi-target immunohistochemical staining on paraffin sections in a diagnostic and/or pathological analysis of human diseases. For example, GAD67 and parvalbumin expression have been studied extensively in the postmortem brains of patients with psychiatric disorders, a system-wide co-localization analysis could provide more information to understand the role GABAergic inhibitory neurons in the pathophysiology of mental illnesses. Finally, since our method can readily identify hundreds of differentially expressed cells across a whole tissue section, a statistical analysis of these cells could easily be performed between different groups. Quantification of the relative expression of each protein in a single cell may also be feasible after being normalized against the average expression level from a large number of other cells. With the development of sophisticated computer software, system-wide analysis of differential expression and co-localization could be conducted more efficiently in the future.

During the preparation of our manuscript, we found that a similar method for sequential chromogenic immunohistochemical staining was reported in literature [4]. However, a completely different antibody stripping method was described. An oxidizing acidic solution containing KMnO_4_/H_2_SO_4_ was used to remove primary antibodies [7]. Another stripping method using Glycine/SDS was also reported [5]. In our comparison studies, however, neither of the two methods generated successful immunostaining of parvalbumin. It remained to be investigated whether the failure was specific to parvalbumin. Conceivably, each stripping method could be limited by its potential detrimental modifications to certain antigens. Different methods, therefore, could complement each other to expand our capabilities in chromogenic immunohistochemical analysis.

## Methods and Materials

### Animals

Mice with mixed S129/Black Swiss background were housed in a climate-controlled animal colony with a reversed day/night cycle. Food (Harlan Teklab, Madison, WI) and water were available *ad libitum*. All procedures were approved by the UCSD Animal Care and Use Committee (permit number: A3033-01) prior to the onset of the experiments. Mice were maintained in American Association for Accreditation of Laboratory Animal Care approved animal facility. This facility meets all Federal and State requirements for animal care.

### Paraffin brain sections

Adult mice were anesthetized with carbon dioxide, and perfused transcardially with 2% PBS buffered paraformaldehyde. Mouse brains were dissected out and further fixed in 4% paraformaldehyde solution at 4°C for 24 hrs. After fixation, the brains were dehydrated and embedded in paraffin [8]. Serial, sagittal sections of the brains were cut at 5 micron thickness.

### The first immunohistochemical stain

The paraffin brain sections were first baked at 60°C oven for one hour to firmly attach the sections to glass slide. After baking, the brain sections were deparaffinized in 3 changes of Xylenes solution for 5 min each, and rehydrated in series of graded ethanol solution (100%, 100%, 95%, 70%, 50%) for 5 min each. After rehydration, the sections were rinsed in distilled water twice, 5 minutes each. The sections were submerged in Tris-EDTA Buffer (10 mM Tris Base, 1 mM EDTA Solution, 0.05% Tween 20, pH 9.0), and autoclaved (Tuttnauer, 2340 M) for antigen retrieval at 121°C for 20 minutes. After autoclaving, the slides were taken out and let to cool down for 20 minutes at room temperature. After a wash in 1× PBS for 5 minutes, endogenous peroxidase activity was quenched with incubation of 0.3% hydrogen peroxide in PBS for 30 minutes at room temperature. The slides were washed twice in 1× PBS, 5 min each time, and blocked with 2.5% Normal Horse Serum (ImmPRESS, Vector Labs, S-2012) at room temperature for 30 minutes. Mouse monoclonal anti-GAD67 antibody (Sigma-Aldrich, G5419), diluted 1∶60K with antibody diluents (Dako, S3022), was used as the primary antibody for overnight incubation at 4°C. Next day, the slides were washed 3 times in PBST (1× PBS/0.1% Tween 20), 5 min each, and further rinsed in 1× PBS for 5 minutes. ImmPRESS peroxidase-micropolymer conjugated horse anti-mouse IgG (Vector Labs, MP-7402) was used as the secondary antibody for 40 minute incubation at room temperature. The slides were then washed three times in PBST (1× PBS/0.1% Tween 20), 5 min each, and further rinsed in 1× PBS for 5 minutes. Chromogenic reaction was conducted with ImmPACT NovaRED Peroxidase Substrate (Vector Labs, SK-4805) for 5 minutes with rotational shaking. After staining, the slides were washed in running tap water for 5 minutes, and air-dried overnight. Next day, the slides were mounted with Cytoseal 60 mounting medium (Richard-Allan Scientific, 8310-16).

### Image scan

The slides were scanned with Aperio ScanScope Model CS Type GL. After calibration with a blank slide, each stained slide was scanned with 200× magnification. The image resolution was at 0.5 µm/pixel. The image resolution could be increased to 0.25 µm/pixel, if the slides were double scanned. The high resolution scan across whole tissue sections provided a system-wide record of protein expression and localization.

### Antibody stripping

The slides were submerged in Xylene to remove coverslip and mounting medium, and further rehydrated in ethanol gradient solution (100%, 100%, 95%, 70%, 50%), 5 minutes each. Antigen retrieval was conducted according to each antigen-specific retrieval protocol. For parvalbumin staining, we used the same condition for both GAD67 and parvalbumin. The sections were submerged in Tris-EDTA Buffer (10 mM Tris Base, 1 mM EDTA Solution, 0.05% Tween 20, pH 9.0), and autoclaved for antigen retrieval at 121°C for 20 minutes. After autoclaving, the slides were taken out and let to cool down for 20 minutes at room temperature. To completely strip the primary antibody, the slides were incubated in 50 ml stripping buffer (10 ml 10% SDS, 3.125 ml 1 M Tris HCl pH 7.5, 400 ul β-mercaptoethanol, and 36.475 ml H2O) for 2 hours at 50°C with gentle shaking. After stripping, the slides were extensively washed in running water for 30 minutes, and a brief rinse in 50% ethanol to completely remove β-mercaptoethanol. Wash slides twice with 1×PBS, 5 minutes each. To test the completeness of antibody removal, the slides were blocked with 2.5% Normal Horse Serum and further incubated with the secondary antibody (ImmPRESS peroxidase-micropolymer conjugated horse anti-mouse IgG). The chromogenic reaction was conducted after washing as described above. No red color stain was observed, suggesting a complete removal of the primary antibody.

### The second, third, fourth, and fifth immunohistochemical staining

After the removal of the first primary antibody (anti-GAD67), the slides were used for immunohistochemical staining with the next primary antibody, anti-parvalbumin. The slides were first blocked with 2.5% Normal Horse Serum, and then incubated with mouse monoclonal anti-parvalbumin antibody (Sigma-Aldrich, p3088) diluted at 1∶4000. After overnight incubation at 4°C, the slides were washed in PBST, and incubated with the secondary antibody, and finally for chromogenic reaction as described above. After mounted, the slides were scanned with Aperio ScanScope. The third, fourth, and fifth rounds of immunostaining were conducted accordingly. Rabbit polyclonal anti-calretinin (Sigma-Aldrich, C7479), anti-Iba-1 (Wako, 019-19741) and anti-MBP were used with dilution at 1∶2000, 1∶2000 and 1∶6000 respectively.

### Image analysis

Images were processed with either Image J or Photoshop. The color of either anti-GAD67 or anti-parvalbumin images was inverted to generate bright positive signal against a dark background, and superimposed onto the other image for differential expression and co-localization analysis. To highlight the cells expressing co-localized GAD67 and parvalbumin proteins, the color of each image was inverted and replaced with either green or red color against a dark background. The co-localization between green and red colors produced bright yellow signal against the dark background in superimposed images. For the co-localization analysis of GAD67, parvalbumin, and calretinin, each image was pseudocolored into green, red and purple.

## Supporting Information

Figure S1 **Co-localization between GAD67 and parvalbumin in the superficial layers of mouse cortex.** Cells expressing only GAD67 appeared as bright spots (black arrow heads). Neurons expressing both GAD67 and parvalbumin were marked with red arrow heads.
(TIF)

Figure S2 **Comparison between different stripping methods.** Our stripping protocol was compared with two stripping methods using either KMnO4 or Glycine/SDS. Three slides were stained well with anti-GAD67 in the first round. The GAD67 staining was then stripped according to each protocol, and the slides were further incubated with the peroxidase-conjugated secondary antibody and finally stained with NOVA Red substrate in the second round. All three methods were effective in stripping because of the absence of significant staining. The slides were further stripped for immunohistochemical analysis of parvalbumin in the third round. Our protocol generated strong immunostaining signal for parvalbumin, however, no signal was observed from the slides stripped with either KMnO4 or Glycine/SDS.
(TIF)

Figure S3 **Distinct expression patterns of parvalbumin and calretinin.** The images of parvalbumin and calretinin were pseudocolored into red and purple, and further superimposed together. Four white arrows pointed to 4 calretinin expressing neurons which did not express parvalbumin. Across the section, we did not observe any co-localization between parvalbumin and calretinin in mouse cortex.
(TIF)
