# Supplementary figures and images for: System-Wide Immunohistochemical Analysis of Protein Co-Localization
Source: PLoS One. 2012 Feb 21;7(2):e32043. doi: 10.1371/journal.pone.0032043 (PMC3283725; doi:10.1371/journal.pone.0032043)

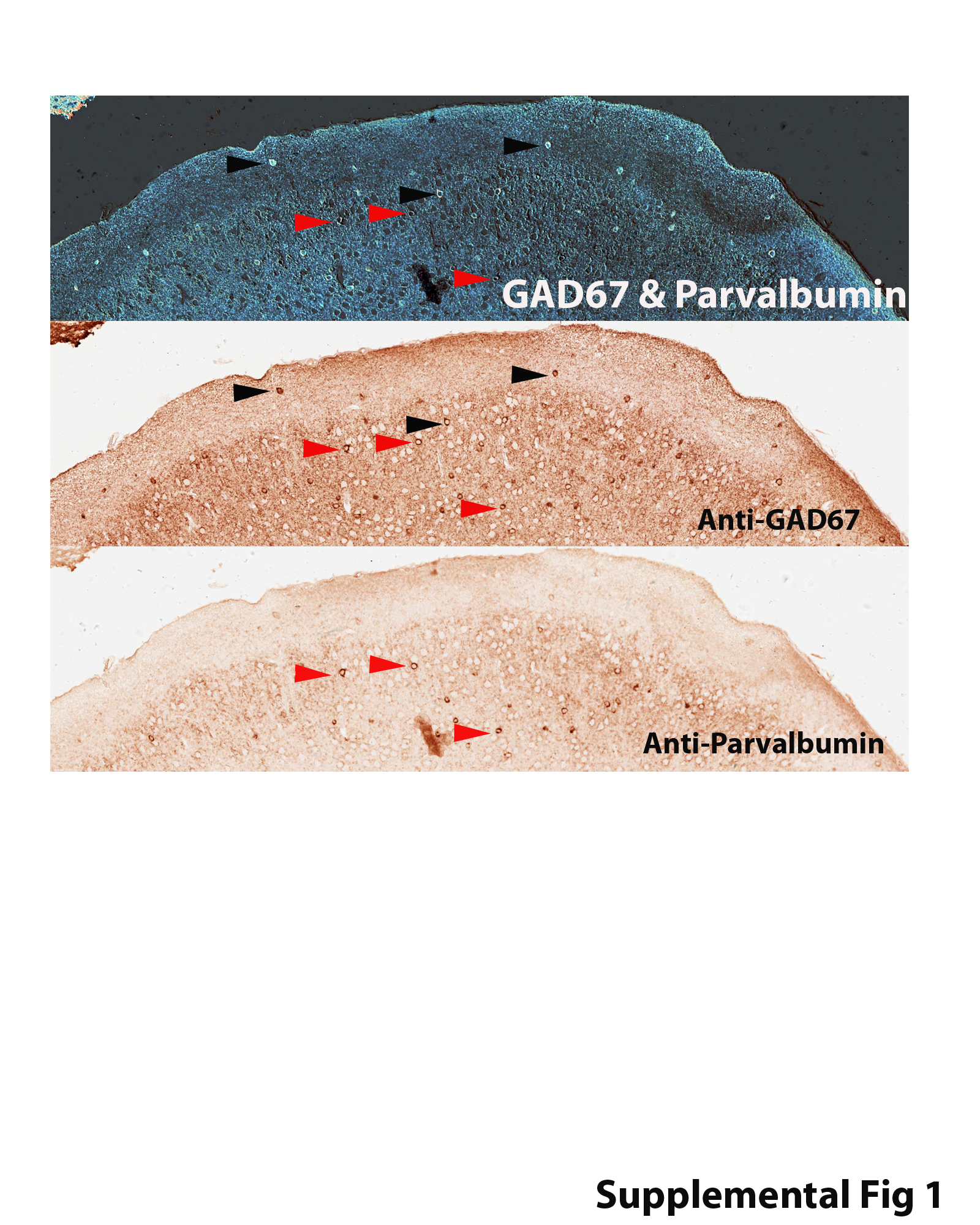

Supplement: Figure S1 — Co-localization between GAD67 and parvalbumin in the superficial layers of mouse cortex. Cells expressing only GAD67 appeared as bright spots (black arrow heads). Neurons expressing both GAD67 and parvalbumin were marked with red arrow heads. (TIF) [file pone.0032043.s001.tif]

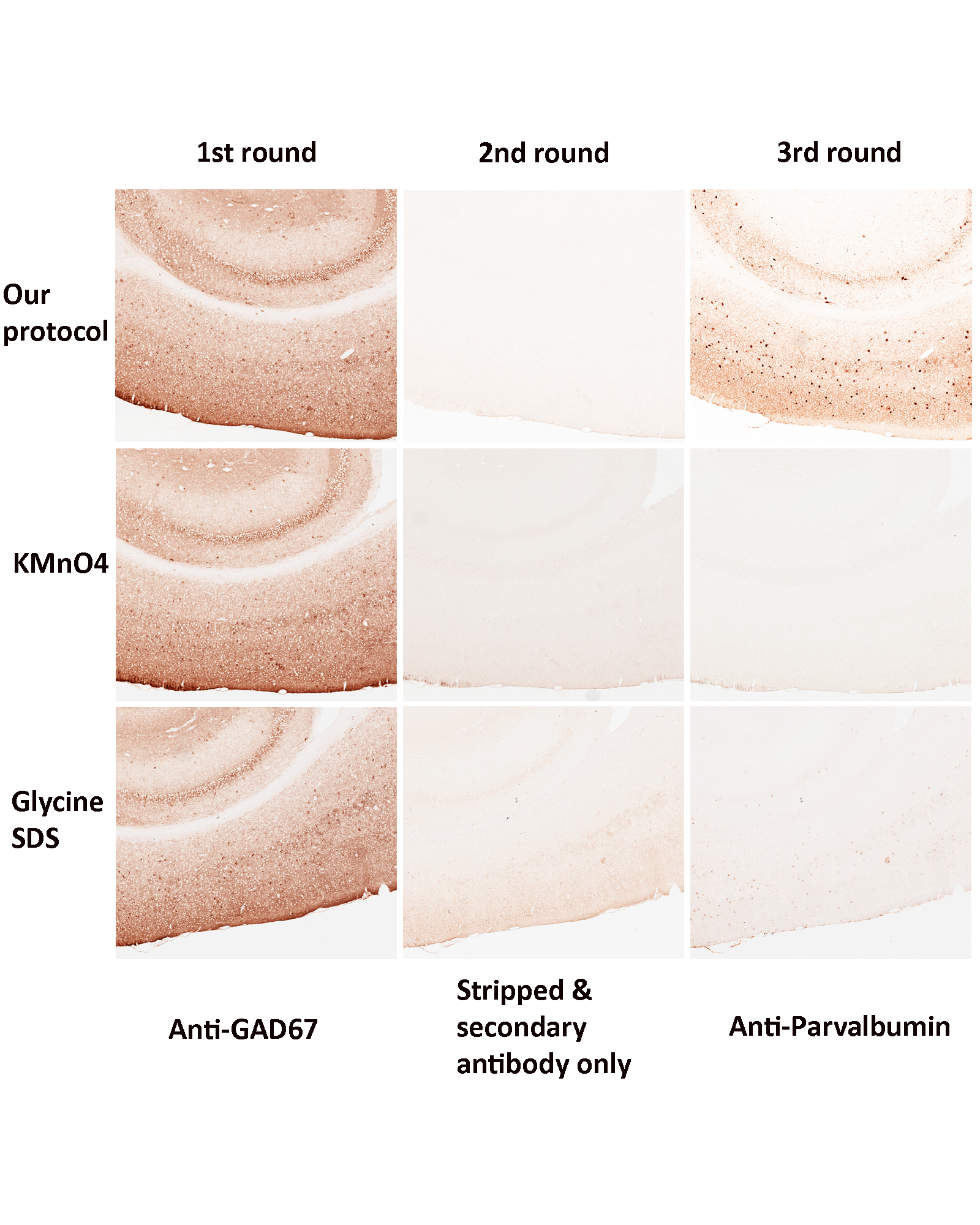

Supplement: Figure S2 — Comparison between different stripping methods. Our stripping protocol was compared with two stripping methods using either KMnO4 or Glycine/SDS. Three slides were stained well with anti-GAD67 in the first round. The GAD67 staining was then stripped according to each protocol, and the slides were further incubated with the peroxidase-conjugated secondary antibody and finally stained with NOVA Red substrate in the second round. All three methods were effective in stripping because of the absence of significant staining. The slides were further stripped for immunohistochemical analysis of parvalbumin in the third round. Our protocol generated strong immunostaining signal for parvalbumin, however, no signal was observed from the slides stripped with either KMnO4 or Glycine/SDS. (TIF) [file pone.0032043.s002.tif]

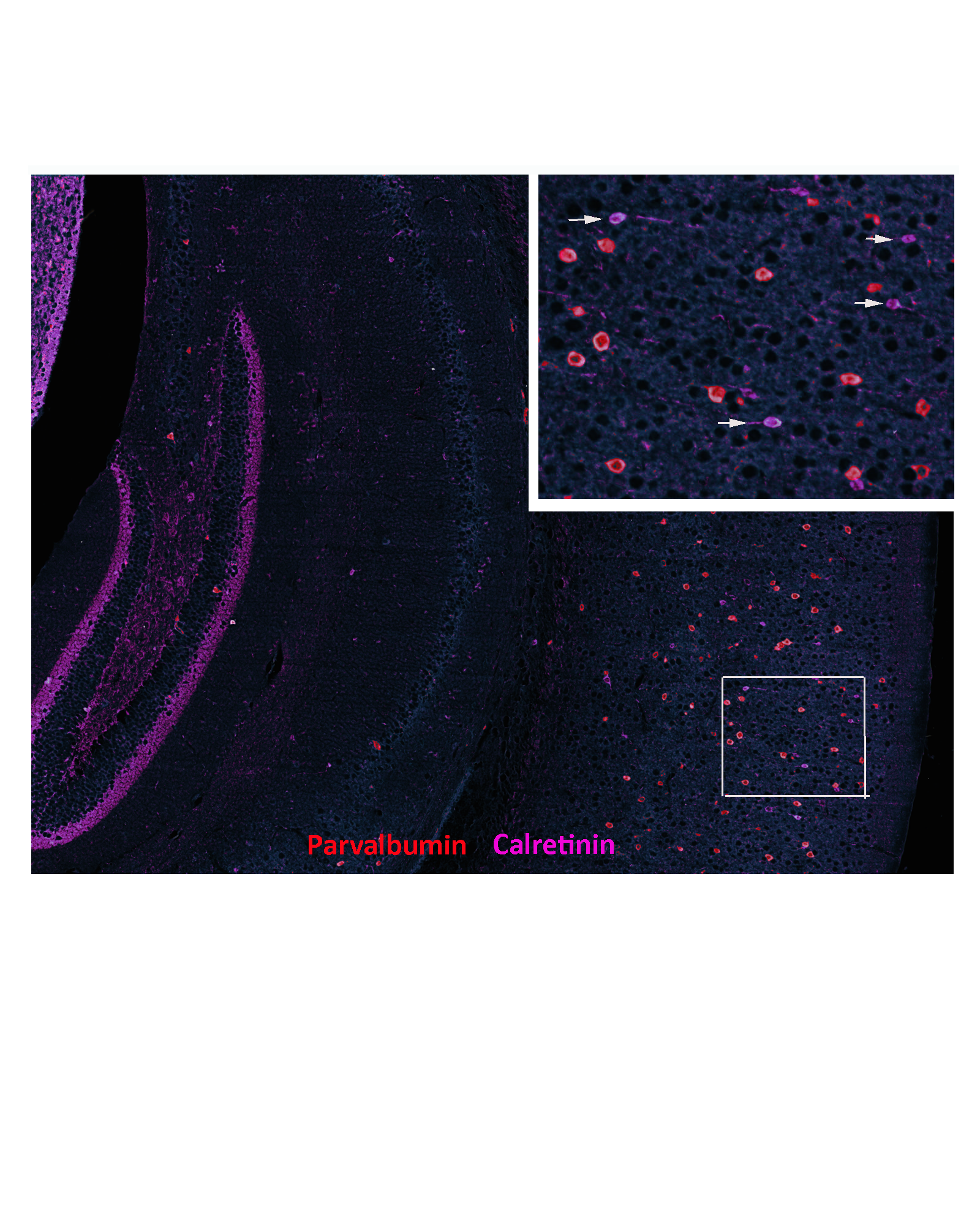

Supplement: Figure S3 — Distinct expression patterns of parvalbumin and calretinin. The images of parvalbumin and calretinin were pseudocolored into red and purple, and further superimposed together. Four white arrows pointed to 4 calretinin expressing neurons which did not express parvalbumin. Across the section, we did not observe any co-localization between parvalbumin and calretinin in mouse cortex. (TIF) [file pone.0032043.s003.tif]
